# A Deep Learning-Based Method for Bearing Fault Diagnosis with Few-Shot Learning

**DOI:** 10.3390/s24237516

**Published:** 2024-11-25

**Authors:** Yang Li, Xiaojiao Gu, Yonghe Wei

**Affiliations:** College of Mechanical Engineering, Shenyang Ligong University, Nanping Middle Road 6, Shenyang 110159, China; 18340380986@163.com (Y.L.); 18804033860@163.com (X.G.)

**Keywords:** KANs, CNN, small sample, fault diagnosis, diffusion network, bearing, tool

## Abstract

To tackle the issue of limited sample data in small sample fault diagnosis for rolling bearings using deep learning, we propose a fault diagnosis method that integrates a KANs-CNN network. Initially, the raw vibration signals are converted into two-dimensional time-frequency images via a continuous wavelet transform. Next, Using CNN combined with KANs for feature extraction, the nonlinear activation of KANs helps extract deep and complex features from the data. After the output of CNN-KANs, an FAN network module is added. The FAN module can employ various feature aggregation strategies, such as weighted averaging, max pooling, addition aggregation, etc., to combine information from multiple feature levels. To further tackle the small sample issue, data generation is performed on the original data through diffusion networks under conditions of fewer samples for bearings and tools, thereby increasing the sample size of the dataset and enhancing fault diagnosis accuracy. Experimental results demonstrate that, under small sample conditions, this method achieves higher accuracy compared to other approaches.

## 1. Introduction

In modern industry, the widespread adoption of “smart manufacturing” and advancements in high-end manufacturing technologies have highlighted the increasing importance of strengthening machinery health management [1] to achieve system intelligence. Rolling bearings are key components in many transmission systems, typically operating under high loads and at high speeds. Any failure can significantly reduce the efficiency of mechanical equipment, potentially leading to substantial economic losses and safety accidents. Therefore, developing efficient bearing fault diagnosis technologies is crucial, as it not only helps reduce economic costs but also prevents potential accidents from occurring.

Bearing fault diagnosis research is progressively dependent on machine learning and deep learning technologies [2]. These techniques have advantages in feature extraction and pattern recognition, making them effective tools to overcome the limitations of traditional methods. Deep learning models, with their powerful nonlinear fitting ability, can automatically extract deep features from complex vibration signals, effectively compensating for the impact of insufficient sample sizes.

In recent years, data-driven approaches using deep learning have gained significant attention, with projects such as robot gearbox fault diagnosis [3] demonstrating the capabilities of deep learning in fault detection. In the field of machine learning, projects such as RV gearbox fault diagnosis [4] have showcased the powerful fault detection capabilities of machine learning by utilizing information obtained from motor current feature analysis for feature extraction, selection, and reduction. Therefore, machine learning and deep learning play a central role in bearing fault diagnosis. Despite significant progress in these technologies, further optimization and validation are still required in practical applications to ensure their reliability and feasibility in complex industrial environments.

Currently, bearing fault diagnosis faces significant challenges due to the Few-shot size [5]. The difficulty of fault data acquisition and the limited number of samples make it challenging for traditional diagnostic methods to effectively extract fault features under small sample conditions, resulting in low diagnostic accuracy and affecting the reliability and timeliness of fault detection. As a result, enhancing diagnostic accuracy with limited samples has emerged as a critical challenge.

On the one hand, Fratti, R et al. [6] proposed transfer learning by applying pre-trained models to tool fault diagnosis, making full use of the abundant existing data. In a robotic component project [7], researchers have applied transfer learning to bearing fault detection, highlighting its ability to generalize across different operating conditions. On the other hand, Gama, P.H.T. et al. [8] utilized meta-learning to acquire prior knowledge for small-sample fault diagnosis. Additionally, Shorter, C. et al. recommended using data augmentation to increase the sample size, thereby achieving accurate fault diagnosis by expanding the dataset. In a bearing fault diagnosis project, Liao et al. [9] introduced a novel bearing fault diagnosis framework named DTM-Bearing, which improves the DDPM network and achieves accurate classification of bearing faults.

Recently, Liu et al. [10] emphasized in their groundbreaking study that Kolmogorov–Arnold Networks (KANs) can address several inherent limitations of multilayer perceptrons (MLPs), especially in handling complex function mappings within high-dimensional spaces. KANs, grounded in the Kolmogorov extension theorem [11] and Arnold’s work [12], provide a neural network structure that theoretically supports the representation of any continuous multivariate function as a combination of univariate functions. By leveraging this structure, KANs decompose complex multidimensional functions into simpler unidimensional functions for resolution, thereby reducing network complexity and training difficulty. Due to their unique decomposition capabilities, KANs excel in high-dimensional data processing and function approximation, demonstrating significant advantages in minimizing overfitting and enhancing generalization. This capability makes KANs highly effective for tasks like image recognition [13,14], object detection [15], and segmentation [16].

Researchers are increasingly investigating the applicability of Kolmogorov–Arnold Networks (KANs) across diverse fields to unlock their potential. The KAN-GPT project [17] utilizes KANs in a generative pre-trained transformer (GPT) for language modeling, highlighting their promise in natural language processing. Similarly, the KAN-mixer project [18] showcases the effectiveness of KAN-based algorithms in visual tasks, introducing natural KAN layers to address gaps in prior research. Han et al. [19] introduced KANs into time series forecasting, benefiting from their superior mathematical properties and interpretability. Rege Cambrin, D et al. [20] integrated KAN layers into the U-Net architecture (U-KAN) using Sentinel-2 and Sentinel-1 satellite images for agricultural field segmentation, analyzing the performance and interpretability of these networks. Similarly, the KANs-based approach in [21] investigates integrating KANs into Transformer architectures by substituting traditional linear layers with KAN layers.

Despite the growing interest in developing various KAN variants related to visual modeling and their corresponding repositories, formal scientific research remains insufficient, mainly focusing on the general applicability of KANs and emerging repositories, particularly for classification tasks. To address this gap, a bearing fault diagnosis model combining KANs, CNNs for feature extraction, and an FAN [22] module for feature fusion has been proposed. The model uses continuous wavelet transform (CWT) to construct time-frequency spectrograms of bearing vibration signals. The KANs-CNN network extends the concept of KANs to convolutional layers, replacing the typical linear transformations in traditional convolutions with learnable nonlinear activation functions at each pixel. Additionally, the DDPM network is used for data augmentation on the raw data. Experiments conducted on the Case Western Reserve University bearing dataset evaluate the model’s performance in terms of accuracy, training efficiency, and model parameters, comparing it with traditional fault diagnosis models. To address the small sample fault diagnosis problem, a diffusion network [23] is employed to augment the original dataset, increasing the sample size and thereby improving diagnostic accuracy, ultimately achieving effective fault diagnosis for small samples in bearings.

## 2. Methodology

**Figure 1:**
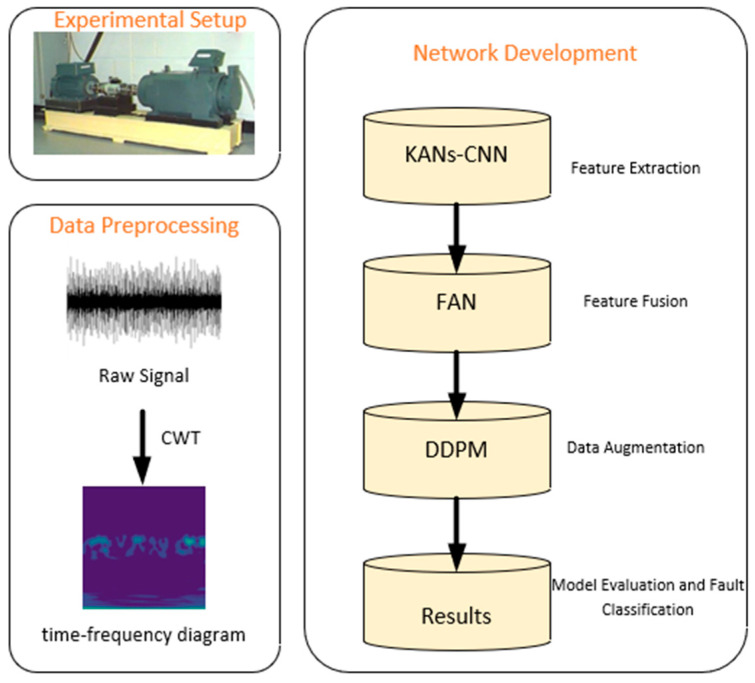
Methodology of the proposed research work.

Figure 1 illustrates the overall process associated with this research. After signal acquisition, the data undergoes preprocessing, including the removal of invalid data, data flushing, and denoising. The original vibration signals are then converted into time-frequency images using a continuous wavelet transform (CWT) to extract more detailed information. The network part mainly focuses on extracting deeper and more complex features through the nonlinear functions of KANs-CNN, and feature fusion is performed using FAN to combine information from multiple feature layers. Data augmentation is then carried out using DDPM to address the small sample issue. Finally, the fault diagnosis results are outputted, and the model is evaluated.

Neural networks are artificial neural network models designed based on the structural and functional characteristics of biological neural systems, possessing strong adaptability and nonlinear mapping capabilities [24]. A neural network consists of multiple neurons (or nodes) that are interconnected through connection weights, forming a multi-layered network structure. Each neuron receives signals from other neurons, linearly combines these signals with weights, and then applies a nonlinear transformation through an activation function before finally outputting to the next layer of neurons or the output layer.

### 2.1. MLP (Multilayer Perceptron)

A multilayer perceptron (MLP) is a type of feedforward neural network and a fundamental structure in artificial neural networks. It typically consists of at least three layers: the input layer, one or more hidden layers, and the output layer. Each node in a layer is connected to nodes in the next layer via weighted connections, and the hidden layer nodes apply activation functions (such as ReLU or Sigmoid) [25] to introduce nonlinearity.

The Universal Approximation Theorem [26] provides a theoretical foundation for the application of multilayer perceptrons (MLPs). According to this theorem, a feedforward neural network with a single hidden layer containing a finite number of neurons can approximate any continuous function on a compact subset of Euclidean space. Mathematically, an MLP with L layers can be represented as follows:

(1) $$f(x)=W_{L}\sigma (W_{L-1}\sigma (\cdots \sigma (W_{2}\sigma (W_{1}x+b_{1})+b_{2})\cdots )+b_{L-1})+b_{L}$$

Here, x is the input vector. $W_{l}$ is the weight matrix of the l-th layer. $b_{l}$ is the corresponding bias vector. $l=1,2,\ldots ,L。\;\sigma \left(\cdot \right)\;$is the activation function, which is usually a nonlinear function. f(x) is the output vector, which is the final prediction result of the model.

The computation of each layer consists of the weighted sum $\left(W_{l}h_{l-1}+b_{l}\right)$ and the activation function (σ(⋅)). The output layer provides the final result through a linear transformation. This structure, through the composition of multiple layers, enables the MLP to approximate complex nonlinear functions.

Training an MLP typically employs the backpropagation algorithm, which minimizes error by adjusting weights. Due to their simple structure and versatility, MLPs are widely used in tasks such as classification [27] and regression [28]. However, compared to more complex deep neural networks, MLPs may exhibit limited performance when handling high-dimensional data or intricate tasks.

### 2.2. Differences Between KANs and MLPs

In an MLP, the connections between neurons are usually represented by real-valued weights, which reflect the strength of the connections, while each neuron is associated with a nonlinear activation function. Thus, the computation process of an MLP is as follows: the weighted inputs are summed, and the activation function then introduces non-linearity. The innovative design of KANs lies in that it does not represent weight parameters as real numbers; instead, it replaces them with a B-spline function [29] that directly connects two neurons, replacing the linear weights in MLPs. In other words, KAN neurons are “non-perceptive”, as they simply “aggregate” the function outputs of the connections (performing a simple summation). The activation function is moved from the nodes to the edges, which means that the computation order changes to “transform first and then add.

**Figure 2:**
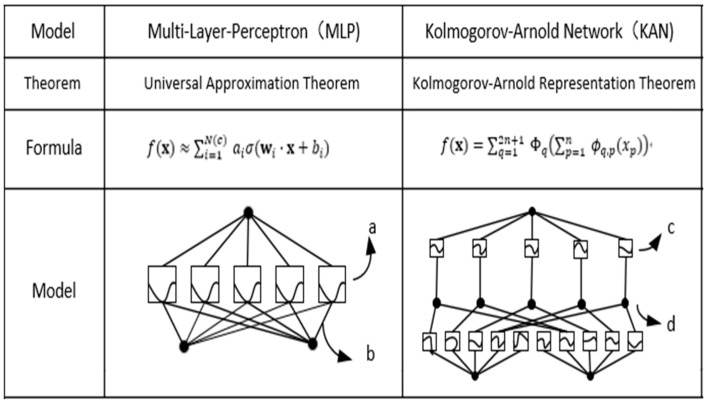
The detailed differences between MLP and KAN.

In summary, unlike MLPs, which have fixed activation functions at the nodes, KANs use learnable activation functions at the edges, as shown in Figure 2.

The letters in the figure represent (a) fixed activation functions at the nodes, (b) learnable weights on the edges, (c) learnable activation functions on the edges, and (d) operations at the nodes.

The MLP formula is

(2) $$f(x)=\sum_{i=1}^{N_{c}}\;a_{i}\sigma (w_{i}\cdot x+b_{i})$$

Here, f(x) is the input function and represents the predicted value of the neural network for the input x. x is the input vector, representing the input data to the network. $N_{c}$ is the number of neurons, indicating the total number of neurons in that layer. i is the index of the neuron, representing the i-th neuron. $a_{i}$ is the weight coefficient, representing the weight connected to the i-th neuron. σ is the activation function. $w_{i}$ is the weight vector, representing the input weights associated with the i-th neuron. $b_{i}$ is the bias, representing the offset for each neuron, used to adjust the input to the activation function.

The KAN formula is

(3) $$f(x)=\sum_{q=1}^{2n+1}\;\Phi_{q}(\sum_{p=1}^{n}\;\phi_{q,p}(x_{p}))$$

Here, f(x) is the input function, and represents the predicted value of the neural network for the input x. x is the input vector, representing the input data to the network. q represents the index of the activation function, ranging from 1 to 2n + 1, which is the total number of nonlinear activation functions. $\Phi_{q}$ is the q-th learnable nonlinear activation function, which maps the weighted sum of the inputs to the output. p is the index representing the input dimensions. n represents the number of input dimensions, which is the total dimensionality of the input vector x. $\phi_{q,p}$ is typically a B-spline function, used for nonlinear transformation of the input data. $x_{p}$ represents the p-nd input feature.

Therefore, KANs fundamentally do not have linear weight matrices; instead, each weight parameter is replaced by a learnable one-dimensional function, parameterized as a spline function. The nodes in the KANs simply perform a summation of the incoming signals without applying any nonlinearity.

### 2.3. KANs (Kolmogorov–Arnold Networks)

#### 2.3.1. The Kolmogorov–Arnold Representation Theorem

The Kolmogorov–Arnold representation theorem [30] asserts that any continuous multivariate function can be represented as a combination of univariate functions. This theory was established by Soviet mathematician Andrey Kolmogorov in the 1950s. He proved that in integrable systems (whose motion can be described by a set of action-angle variables) many quasi-periodic motions can still be preserved when subjected to sufficiently small perturbations. Kolmogorov’s student, Vladimir Arnold, further expanded this research, validating this result in a broader context.

If f is a multivariate continuous function on a bounded domain, then f can be expressed as a finite combination of continuous functions through composition and binary operations of addition. In other words, any continuous function $f\left(x_{1},\ldots ,x_{n}\right)$ can be expressed as a finite nested combination of univariate functions, as represented by the following formula.

(4) $$f(x)=\sum_{q=1}^{2n+1}\;\Phi_{q}\left(\sum_{p=1}^{n}\;\varphi_{q,p}\left(x_{p}\right)\right)$$

where $\Phi_{q}$ and $\varphi_{q,p}$ are both univariate functions.

More specifically, for a smooth $f:[0,1{]}^{n}\to R$, as shown in the following formula.

(5) $$f(x)=f\left(x_{1},\cdots ,x_{n}\right)=\sum_{q=1}^{2n+1}\;\Phi_{q}\left(\sum_{p=1}^{n}\;\phi_{q,p}\left(x_{p}\right)\right)$$

Here, $x_{p}$ represents the p-rd element of the vector x, so the range of p is from 1 to n (where n is the dimensionality of the input vector). The index q is used to iterate over each component of the external function Φ.

Thus, the univariate function $\phi_{q,p}$ processes the p-rd component of the input vector x, and the summation of the q-th external function contributes a term. The theorem states that you can use 2n + 12n + 12n + 1 external functions—each represented by $\Phi_{q}$, which is a univariate function (which acts on the sum composed of the outputs of the internal univariate function $\phi_{q,p}$) that represents any multivariate function f. In KANs, such a structure enhances the model’s flexibility, particularly by introducing adjustable basis functions (spline functions), enabling the model to freely adjust the connection strengths and shapes between nodes for a more efficient function approximation.

#### 2.3.2. B-Spline (Basis Spline) Functions

As previously mentioned, the innovative design of KAN lies in its weight parameters being represented not as real numbers but as B-spline functions, which directly connect two neurons and replace the linear weights found in MLPs. To better understand this, this chapter will provide additional insights into the principles and functions of spline functions.

A spline function is a smooth function used for approximating or interpolating data, composed of piecewise polynomials that maintain certain continuity at the joints. This design allows for precise approximation or interpolation of data through combinations of piecewise polynomials, providing greater flexibility in function representation without sacrificing smoothness.

**Figure 3:**
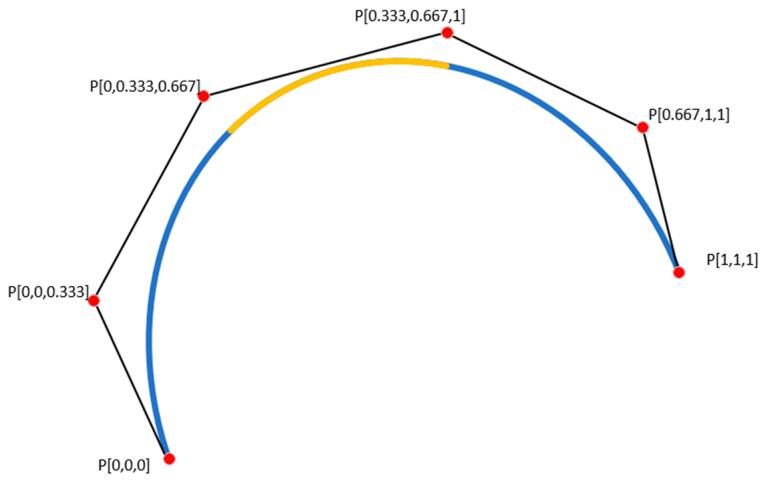
A schematic representation of a spline curve.

As shown in Figure 3, in a B-spline function, all points P on the curve are called control points (or grid points). The B-spline function ensures smoothness, particularly during transitions between control points, preventing sharp angles or discontinuities. Each control point influences the shape of the curve at that point through a linear combination of spline polynomials.

The yellow part represents the fitted path of the spline curve (B-Spline), highlighting the smooth transitions between control points as well as the continuity and flexibility of the spline function. The blue part consists of lines connecting the control points, illustrating their positions and influence on the shape of the curve. Overall, the yellow curve demonstrates the smooth interpolation characteristics of the B-Spline, while the blue lines help to understand the relationship between the control points and the curve.

B-splines are an interpolation method constructed by connecting piecewise polynomials. Each segment’s polynomial is determined by a set of control points, which maintain a certain continuity between them to ensure the smoothness of the function. This design allows B-splines to flexibly approximate or interpolate data while preserving the overall shape.

**Figure 4:**
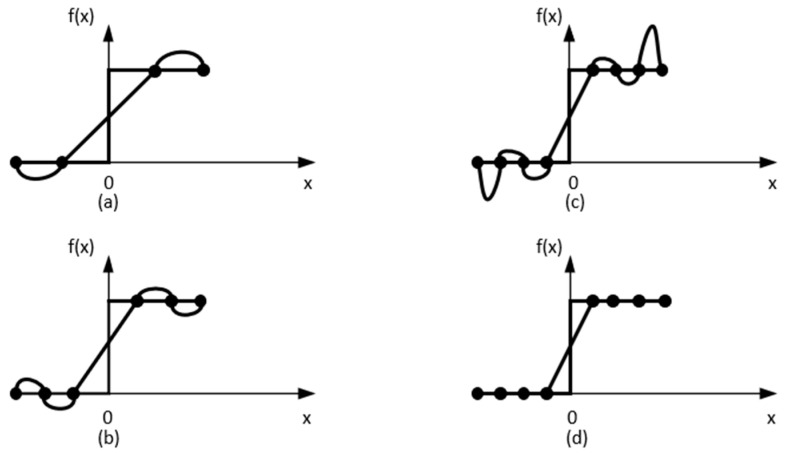
Spline interpolation, showcasing different orders of spline interpolation. (**a**) for cubic interpolation, (**b**) for 7th-order interpolation, (**c**) for 5th-order interpolation, and (**d**) for linear spline.

As shown in Figure 4, f(x) represents the interpolation function: (a) for cubic interpolation, (b) for 7th-order interpolation, (c) for 5th-order interpolation, and (d) for linear spline. Among these, the linear spline has the lowest order, connecting control points directly with linear functions. These different orders of splines indicate the degree of smoothness in interpolation. The higher the order, the smoother the transition of the interpolation function between control points, although the computational complexity also increases. KAN utilizes these spline functions to represent complex nonlinear mappings as a combination of piecewise polynomials, thereby enhancing the model’s expressiveness when handling continuous functions.

For B-splines, their polynomial expressions can be represented using the Cox-de Boor recursion formula.

(6) $$\begin{array}{l} B_{i,0}(x):=\left\{\begin{array}{cc} 1 & \mathrm{if}\;t_{i}\leq x<t_{i+1}\\ 0 & \mathrm{otherwise}. \end{array}\right.\\ B_{i,k}(x):=\frac{x-t_{i}}{t_{i+k}-t_{i}}B_{i,k-1}(x)+\frac{t_{i+k+1}-x}{t_{i+k+1}-t_{i+1}}B_{i+1,k-1}(x) \end{array}$$

where $B_{i,0}(x)$ is the B-spline function. i is the index of the spline. It represents the position of the current spline segment within the piecewise interval. K is the order of the B-spline, representing the polynomial degree of the spline. x is the independent variable, representing the input value. $t_{i}$ is the knot value, representing the segmented control points of the spline function. $\frac{x-t_{i}}{t_{i+k}-t_{i}}$: This is one of the weight coefficients in the recursive computation, used to weight the B-splines between knots, ensuring a smooth transition. $\frac{t_{i+k+1}-x}{t_{i+k+1}-t_{i+1}}$ is another weight coefficient in the recursive computation that works in conjunction with the previous coefficient to ensure a smooth connection of the B-spline.

#### 2.3.3. The KAN Structure

KAN enhances the Kolmogorov–Arnold representation by using learnable activation functions on the graph edges. A KAN layer with inputs and outputs is defined as follows:

(7) $$\Phi =\left\{\phi_{q,p}\right\},p=1,2,\ldots ,n_{in},q=1,2,\ldots ,n_{out}$$

where $\phi_{q,p}$ is the learnable function, parameterized as a spline.

(8) $$x_{l+1}=\sum_{i=1}^{n_{l}}\;\phi_{l,j,i}(x_{l,i}),j=1,\ldots ,n_{l+1}$$

In matrix form, it can be represented as:

(9) $$x_{l+1}=\Phi_{l}x_{l},$$

where $\Phi_{l}$ corresponds to the function matrix of the l-th layer of the KAN, which is greater than that of the MLP. In particular, the approximation bound of the KAN is:

(10) $${∥f-\left(\Phi_{L-1}^{G}\circ \cdots \circ \Phi_{0}^{G}\right)x∥}_{C^{m}}\leq CG^{-k-1+m}$$

where G is the grid size, k is the degree of the B-spline, and C is a constant. ΦGL−1···ΦG0 represents the composition of function matrices from layer 0 to layer L−1. The composition operation indicates that the output of one function matrix is used as the input for the next function.

#### 2.3.4. CNNs (Convolutional Neural Networks)

A typical CNN structure includes the following components: input layer, which takes in raw data like images; the convolutional layer, which extracts local features; the pooling layer, which reduces dimensionality while retaining key features; the fully connected layer, which performs the final classification or regression task; and the output layer, which produces the results, such as classification labels.

The convolution operation is the core of CNNs, involving element-wise multiplication and summation of the input data with convolutional kernels to generate feature maps [31]. The formula is as follows:

(11) $$\left(f*g)(t)=\sum_{\tau =-\infty }^{\infty }\;f(\tau )\cdot g(t-\tau \right)$$

where (f∗g)(t) represents the symbolic notation for the convolution operation, f represents the input signal or input data, such as an image or a time series, and g(t−τ) represents the flipping and shifting of the convolutional kernel. In signal processing or neural networks, convolution is used to combine the input signal with a convolutional kernel to extract features.

### 2.4. Fault Diagnosis Model Based on KANs-CNN

#### 2.4.1. Conv KANs

The key concept behind combining KANs with CNNs is to replace the traditional linear activation functions with learnable activation kernels. For simplicity, the convolutional layer based on KAN is called the KAN Conv layer, where the convolution kernel is defined as:

(12) $$Conv\;KAN\;Kernel=\left[\begin{array}{cc} \phi_{11} & \phi_{12}\\ \phi_{21} & \phi_{22} \end{array}\right]$$

where $\left[\begin{array}{cc} \phi_{11} & \phi_{12}\\ \phi_{21} & \phi_{22} \end{array}\right]$ represents the specific numerical elements in the convolutional kernel. These values are used in pointwise multiplication with the corresponding parts of the input data during the convolution process to help extract features.

In Conv KANs (Convolutional KANs), the KAN concept is applied to the convolution operation by using learnable B-spline basis functions instead of the traditional convolution method. The formula for the KAN-based convolution kernel is:

(13) $$y_{i,j}=\sum_{m,n}\;\phi_{m,n}(x_{i+m,j+n})$$

Here, $\phi_{m,n}$ is the learnable function based on B-splines.

#### 2.4.2. Details of the Process

(1) KAN Conv layer: Consider a KAN layer with input $x\in R^{n_{in}}$ and output $y\in R^{n_{out}}$. This layer applies a learnable function matrix ϕ:

(14) $$y=\Phi \left(x\right)$$

where $\Phi =\{\phi_{q,p}\}$ and $\phi_{q,p}$ are parameterized B-spline functions.

(2) Where each learnable function φ in the KANs is parameterized as a B-spline given by:

(15) $$\phi (x)=w_{1}\cdot spline(x)+w_{2}\cdot b(x)$$

(3) Structure of the KAN Conv layer: Suppose there is an input $x\in R^{H\times W}$ and a convolution kernel of size K × K at each position (i,j). The result of the convolution operation is calculated using a set of learnable functions (as shown in Equation (14)) Φ. To enable the KAN to handle multi-channel input, the convolution kernel operates on each input channel and generates the corresponding output channel.
(4) Calculation of the convolution kernel: Each KAN Conv kernel has a size of G + 2, where G is the grid size (the degree of the B-spline). Therefore, for each KAN Conv kernel, the total number of parameters is K^2^(G + 2).

This design allows the KAN Conv kernel to adapt to complex nonlinear patterns while maintaining compatibility with traditional CNN architectures, thus improving the convolution operation’s expressive capability.

#### 2.4.3. FAN (Feature Aggregation Network)

FAN (Feature Aggregation Network) is a neural network architecture designed to combine shallow and deep features, thereby obtaining unique aggregated features. The network merges layers at similar stages and feeds their features into previous layers to capture hierarchical information more effectively. Additionally, to avoid dimensionality reduction, shallow layer features are directly integrated into the final feature map through skip connections. This approach preserves important fine-grained information while allowing deeper features to provide abstract representations, improving the network’s overall feature extraction ability.

In FAN, assuming the feature map of the shallow layer is $F_{s}$ and the feature map of the deep layer is $F_{d}$, they can be aggregated using the following formula:

(16) $$F_{\mathrm{aggregated}}=F_{d}+\alpha \cdot F_{s}$$

Here, α is a scaling parameter used to balance the contribution of shallow and deep features in the aggregated feature.

To better understand the feature aggregation process, further clarification is provided here.

Multi-layer feature aggregation: Suppose there are multiple shallow feature maps $F_{s1},F_{s2},\ldots ,F_{sn}$ and one deep feature map $F_{d}$. Multi-layer feature aggregation can be performed through a weighted sum:

(17) $$F_{\mathrm{aggregated}}=F_{d}+\sum_{i=1}^{n}\;\alpha_{i}\cdot F_{si}$$

Here, $\alpha_{i}$ is the weight of each shallow feature map, which controls the importance of each layer’s features in the aggregated feature.

Skip connection fusion: To avoid the loss of detailed information in shallow features during transmission, skip connections can be used to fuse shallow and deep features. Suppose $F_{skip}$ is the feature passed through the skip connection, then the final output feature map $F_{output}$ can be expressed as:

(18) $$F_{output}=ReLU(W\cdot F_{aggregated}+F_{skip})$$

Here, W is the weight matrix, and ReLU is the activation function used to introduce non-linearity, ensuring that the fused features contain rich multi-level information.

### 2.5. DDPM (Diffusion Network)

The denoising diffusion probabilistic model (DDPM) [32], initially overlooked since its 2020 publication due to its complexity compared to GAN [33], has quickly gained attention, now representing over half of the relevant submissions at the ICRL conference. Currently, the top text-to-image generation models—OpenAI’s DALL·E 2 [34] and Google’s Imagen—are both based on diffusion models.

Diffusion models, inspired by nonequilibrium thermodynamics [35], start by defining a Markov chain for the diffusion steps [36], which progressively adds random noise to the data. The model then learns the reverse diffusion process to generate data samples from the noise. Unlike Variational Autoencoders (VAEs) [37] or flow models, diffusion models follow a fixed process and often use a higher-dimensional latent space zzz, enabling them to perform better in generative tasks.

#### 2.5.1. Forward Noise Addition Process

**Figure 5:**
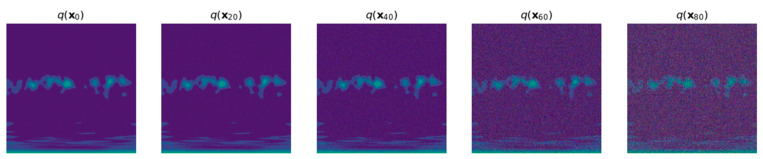
The noise addition process in the diffusion network (displayed every 20 steps).

The forward process, which involves adding noise, is a Markov chain process, as illustrated in Figure 5. The DDPM model consists of two main processes: the forward noise addition (right to left) and the reverse denoising (left to right). In the forward process, Gaussian noise is progressively added to the dataset images [38], while in the reverse process, the added noise is gradually removed to recover the original images.

The noise addition process follows specific mathematical rules, while the denoising process is learned using neural networks. This design enables the neural network to transform random noise into realistic images, facilitating high-quality image generation.

Diffusion models define a probabilistic distribution transformation model T, which can transform the complex distribution $q_{complex}$ formed by the original data $x_{0}$ into a simple prior distribution $p_{prior}$ with known parameters.

(19) $$x_{0}∼q_{complex}⟹T\left(x_{0}\right)∼p_{prior}$$

Specifically, the diffusion model suggests constructing T using a Markov chain by defining a series of conditional probability distributions $q(x_{t}|x_{t-1})$ t ∈ {1,2,3… T}, which sequentially transform $x_{0}$ into $x_{1}$, $x_{2},x_{3}$…$x_{T}$. It is hoped that when T is sufficiently large:

(20) $$T\left(x_{0}\right)∼p_{prior}$$

To simplify and improve efficiency, a Gaussian distribution is used as the prior, making the forward diffusion process equivalent to progressively adding small amounts of Gaussian noise to the samples over T steps.

Specifically, at each step of the Markov chain, Gaussian noise with variance $\beta_{t}$ is added to $x_{t-1}$, resulting in a new latent variable $x_{t}$, which follows the distribution $q(x_{t}|x_{t-1})$. This diffusion process can be expressed as follows:

(21) $$q(x_{t}|x_{t-1})=N(x_{t};\mu_{t}=\sqrt{1-\beta_{t}}x_{t-1},\Sigma_{t}=\beta_{t}I)$$

According to the context, I is the identity matrix, indicating that each dimension has the same standard deviation of $\beta_{t}$. Notably, $q(x_{t}|x_{t-1})$ follows a normal distribution with a mean of μ t and a variance of ∑ t, where ∑ is a diagonal matrix representing the variance (which is $\beta_{t}$ here). Therefore, it is possible to approximate the input from $x_{0}$ to $x_{T}$ in a tractable manner. Mathematically, this posterior probability is defined as follows:

(22) $$q(x_{1:T}|x_{0})=\prod_{t=1}^{T}\;q(x_{t}|x_{t-1})$$

Here, $x_{1:T}$ signifies the repeated application of $q(x_{t}|x_{t-1})$ from time 1 to T. This cumulative multiplication process is overly cumbersome; however, by utilizing the reparameterization trick, we can obtain:

(23) $$x_{t}=\sqrt{1-\beta_{t}}x_{t-1}+\sqrt{\beta_{t}}z_{t-1}$$

(24) $$where\;z_{t-1}\in N(0,I)$$

As β continuously increases, α diminishes over time. Thus, as the forward time progresses, the influence of noise becomes more significant. The noise z follows a Gaussian distribution, and as t approaches positive infinity, $x_{t}$ converges to an isotropic Gaussian distribution. This allows for the direct determination of $x_{t}$ at any given time.

#### 2.5.2. The Reverse Process (Denoising Process)

The reverse process of the Diffusion Model is the inverse of the forward noise addition process, gradually removing noise from the image. Unfortunately, while $q(x_{t}|x_{t-1})$ is known, $q\left(x_{t-1}\left|x_{t},\right.\right)$ remains unknown. However, related research indicates that the reversal of the continuous diffusion process shares the same distributional form as the forward process. That is when the diffusion rate $\beta_{t}$ is sufficiently small and the number of diffusion steps is sufficiently large, the discrete diffusion process approaches the distribution form of the continuous diffusion process $q(x_{t}|x_{t-1})$, which is also a Gaussian distribution, consistent with $q(x_{t-1}|x_{t})$. Nevertheless, $q(x_{t-1}|x_{t})$ cannot be directly obtained; therefore, it is necessary to learn a network model $p_{\theta }\left(x_{t-1}\left|x_{t}\right.\right)$ to fit $q(x_{t-1}|x_{t})$.

(25) $$\begin{array}{c} p_{\theta }\left(x_{t-1}\left|x_{t}\right.\right)=N\left(x_{t-1};\mu_{\theta }\left(x_{t},t\right),\Sigma_{\theta }\left(x_{t},t\right)\right)\\ p_{\theta }\left(x_{0}\right)=\int p_{\theta }\left(x_{0:T}\right)dx_{1:T}\\ p_{\theta }\left(x_{0:T}\right)=p\left(x_{T}\right)\prod_{t=1}^{T}\;p_{\theta }\left(x_{t-1}\left|x_{t}\right.\right) \end{array}$$

In DDPM, the variance is not learned and is set to $\beta_{t}$. Thus, the posterior probability of the Gaussian in the reverse process is defined as:

(26) $$q\left(x_{t-1}\left|x_{t},x_{0}\right.\right)=N\left(x_{t-1};\tilde{\mu }\left(x_{t},x_{0}\right),{\tilde{\beta }}_{t}I\right)$$

The goal in the reverse process is to minimize the distance between the following two Gaussian distributions, which can be achieved by calculating the KL divergence between them, where the mean and variance of $q\left(x_{t-1}\left|x_{t}\right.,x_{0}\right)$ are both known:

(27) $$\begin{array}{cc} q\left(x_{t-1}\left|x_{t}\right.,x_{0}\right) & =N\left(x_{t-1};\tilde{\mu }\left(x_{t},x_{0}\right),{\tilde{\beta }}_{t}I\right)⟷p_{\theta }\left(x_{t-1}\left|x_{t}\right.\right)\\ & =N\left(x_{t-1};\mu_{\theta }\left(x_{t},t\right),\Sigma_{\theta }\left(x_{t},t\right)\right) \end{array}$$

This function acts as the loss function for network training.

### 2.6. Fault Diagnosis Model

#### 2.6.1. Continuous Wavelet Transform (CWT)

The tool vibration signal is first collected from the experimental equipment and presented as a one-dimensional time series. Then, through data preprocessing and continuous wavelet transform (CWT), this one-dimensional vibration signal is converted into a two-dimensional time-frequency diagram.

Specifically, CWT is a signal processing method that enables simultaneous analysis of the signal in both time and frequency domains. Through CWT, we can decompose the original vibration signal into wavelet coefficients at different scales, generating a two-dimensional image that displays the frequency components of the signal as they vary over time. The resulting time-frequency image not only retains the frequency information of the vibration signal but also reflects the dynamic changes in frequency over time. This type of time-frequency image is typically presented as an RGB color image, which facilitates subsequent processing and classification by image-based models.

#### 2.6.2. Fault Diagnosis Process

The KANs-CNN model proposed in this paper aims to enhance the feature extraction capability of small sample data in the context of limited-bearing fault samples, thereby increasing the accuracy of fault diagnosis. KAN utilizes B-spline basis functions for activation, enabling a more precise fitting of complex patterns in the input data, rather than relying solely on linear activation functions. This approach is particularly effective for fault signals that contain significant noise or intricate features.

**Figure 6:**
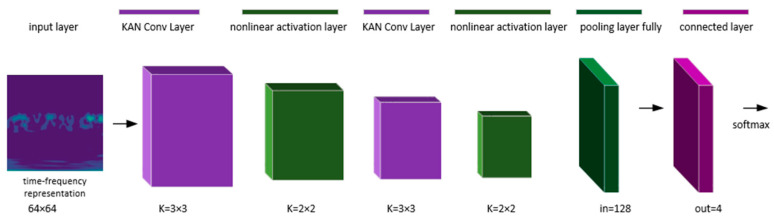
The structure of the KANs-CNN network.

By incorporating KAN, the model’s parameters are simplified compared to traditional convolutional kernels, making it easier to avoid overfitting in small sample scenarios. KAN enhances the model’s ability to distinguish between different types of faults by using learnable activation kernels, which extend the linear representation of the original signal into a complex representation in a nonlinear space. The structure of the KANs-CNN network model is illustrated in Figure 6.

The model is built and trained based on the deep learning framework in Python. The first layer is the input layer, where the input images are time-frequency representations processed through continuous wavelet transform (CWT), with a batch size of 128. This is followed by two KAN convolutional layers (KAN Conv layers) and nonlinear activation layers, where the KAN Conv layers extract local features from the data and, in conjunction with the nonlinear activation functions, further enhance the network’s expressive power.

In KAN, the learnable activation kernel replaces traditional activation functions. The activation kernel Φ can be a B-spline-based function, performing nonlinear transformations after each convolutional layer. The design approach alternates between KAN convolutional layers and nonlinear activation layers twice to enhance the network’s nonlinear expressive capability, capture multi-scale features, and mitigate the vanishing gradient problem. Additionally, Dropout is incorporated into the fully connected layer to prevent model overfitting.

**Figure 7:**
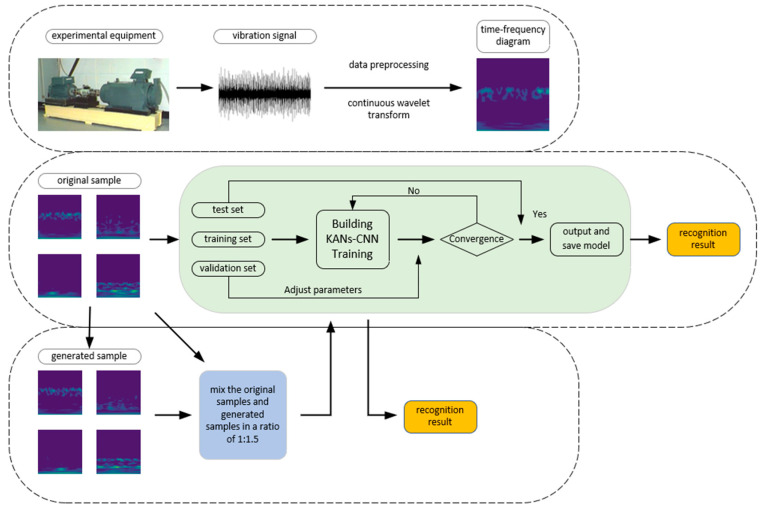
The fault diagnosis flowchart.

To address the issue of limited bearing fault samples, the specific fault diagnosis process is shown in Figure 7. By effectively extracting and analyzing time-frequency features, this method can provide reliable fault identification results even with a limited sample size.

The fault diagnosis process begins with the acquisition of bearing vibration signals, followed by invalid value handling and noise reduction. Fault diagnosis is performed using the KANs network, with DDPM applied for data augmentation and continuously adjusting parameters until the model converges. Once the model converges, it is output and saved for subsequent fault identification and diagnosis.

Additionally, the original data is augmented using a diffusion network, mixing the original samples with generated samples in a 1:1.5 ratio to create a mixed dataset. The KANs-CNN model is then trained on this dataset to achieve higher accuracy. This data augmentation strategy effectively enhances the model’s generalization ability and diagnostic accuracy under small sample conditions.

## 3. Implementation and Results

To comprehensively validate the effectiveness and superiority of the proposed method, a detailed set of experiments was designed. This study selected two representative datasets for experimentation, aiming to thoroughly assess the method’s generalization ability and stability. Additionally, to objectively evaluate the performance of the proposed method, comparative experiments were conducted against the traditional Multi-Layer Perceptron (MLP) and KAN for the fault diagnosis model. For data generation, comparisons were made with classical Generative Adversarial Networks (GANs) and Variational Autoencoders (VAEs) to verify the method’s effectiveness. These comparative experiments will provide important evidence for evaluating the proposed method’s performance in different contexts.

Additionally, comparisons were made with CNN fault diagnosis algorithms that do not utilize generative models, highlighting the advantages of the proposed method in addressing data scarcity and generation capabilities.

In terms of hardware configuration for the experiments, a high-performance computing environment was selected, featuring dual graphics cards and dual GPUs to collaboratively execute the experiments. These configurations provided ample computational resources, ensuring the accuracy and reliability of the results.

During the experiments, all fault diagnosis models were trained for 300 epochs with a learning rate of 0.001 and the Adam optimizer for parameter optimization and model tuning, ensuring proper training and convergence. Python was used as the development language, utilizing the TensorFlow deep learning framework to build and train the models, ensuring smooth execution and accurate results.

### 3.1. Case 1

#### 3.1.1. Experiment Data of Case 1

**Figure 8:**
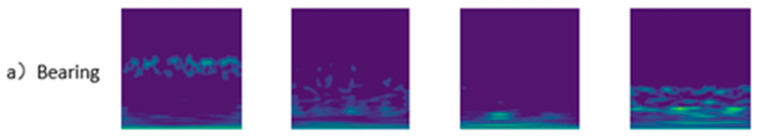
Time-frequency diagram of the bearing after wavelet transform.

The experiment evaluates the proposed method using the bearing dataset from Case Western Reserve University. The setup includes a three-phase induction motor, torque sensor, power testing sensor, and controller. The SKF6205-2RS bearing at the drive end was chosen, with a sampling frequency of 12 kHz and a rotational speed of 1750 rpm. Four types of signals were collected: rolling element fault, inner race fault, outer race fault, and normal condition. Since there was only one normal signal at this speed, one signal was selected, while three signals were collected for each fault condition, resulting in 10 signals in total. After denoising, the continuous wavelet transform was applied to segment and convert the signals into time-frequency representations. A small test set of 60 samples was created, with 18 samples each for rolling element, inner race, and outer race faults, and 6 samples for the normal condition. Additionally, a raw training set of 20 samples, with 10 samples per condition, was labeled as 0, 1, 2, and 3. The dataset was processed into time-frequency images, with examples shown in Figure 8. The description of the CWRU dataset is shown in Table 1.

#### 3.1.2. Experiment Results of Case 1

Fault diagnosis experiments were conducted on the augmented bearing dataset using the KANs-CNN network. The number of samples augmented through DDPM was half of the original samples, and these were combined with the original samples for fault diagnosis using KANs-CNN. The experimental results showed that after only 300 training epochs, the model achieved good convergence, with an average diagnostic accuracy of 98.3% for bearing fault detection.

**Figure 9:**
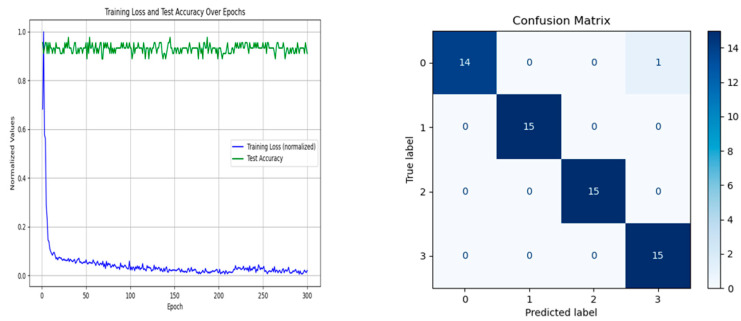
Loss Function, Accuracy, and Confusion Matrix of the KANs-CNN Experiment on the Augmented Bearing Dataset Using DDPM.

These results demonstrate that the KANs-CNN network exhibits excellent classification capability when handling augmented datasets, significantly improving fault diagnosis accuracy. The loss function and confusion matrix are shown in Figure 9.

Fault diagnosis experiments on the bearing dataset were conducted using the KANs-CNN network, with key evaluation metrics presented in Table 2.

**Figure 10:**
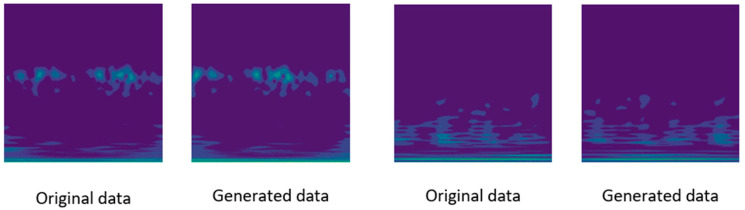
Comparison of time-frequency maps of the bearing dataset before and after using the diffusion network.

On the bearing dataset, the model demonstrated high precision, recall, and F1 scores. Additionally, images generated by the diffusion network (as shown in Figure 10) were added to the original dataset, increasing the sample size. A comparison with the unaugmented dataset was conducted to verify the effectiveness of DDPM in learning fault information from small samples during forward diffusion. During the reverse diffusion process, a large number of samples containing fault information were generated, which aided the fault diagnosis model in extracting fault information, significantly improving the accuracy of fault diagnosis.

Table 2 shows a comparative analysis of the performance of traditional MLP and convolutional KANs with varying layer counts in bearing fault diagnosis. It is evident from the table that the performance of traditional MLP is significantly inferior to that of convolutional neural networks utilizing KAN. As the number of KAN convolutional layers increases, the model’s accuracy, precision, recall, and F1 score gradually improve. With six layers of KAN convolutional networks, the model achieves the highest values for all metrics, regardless of whether the samples are augmented or non-augmented. This indicates that KAN convolutional layers significantly enhance the effectiveness of bearing fault diagnosis.

To evaluate the diffusion network’s effectiveness, a series of experiments, from Experiment 1 to Experiment 15, were conducted. Experiments 1 to 5 used only real data, Experiments 6 to 10 combined real and generated data, and Experiments 11 to 15 relied solely on generated data for fault diagnosis. Detailed results of these experiments are shown in Table 3.

From Experiments 1 to 5, it is evident that as the number of training samples decreases, the model’s accuracy gradually declines. This indicates that, in the absence of additional generated samples, the quantity of samples significantly affects diagnostic accuracy. Experiments 6 to 10 show that, as the number of generated samples increases, the diagnostic accuracy also rises. The highest accuracy is achieved when the ratio of generated to original samples reaches 1:1.5. However, an excess of generated samples does not further improve accuracy, as these samples may contain redundant information, suggesting that generated samples cannot fully replace real data.

Experiments 11 to 15 show that training solely with generated samples, without real data, results in lower accuracy compared to Experiments 1 to 5. This suggests that, while generated samples can approximate the distribution of real data, they have limitations in fault diagnosis. Overall, the combination of generated and real data yields better results than relying on a single data source. This underscores the importance of integrating both generated and actual data to enhance fault diagnosis performance while acknowledging that generated data cannot entirely substitute for real data.

**Figure 11:**
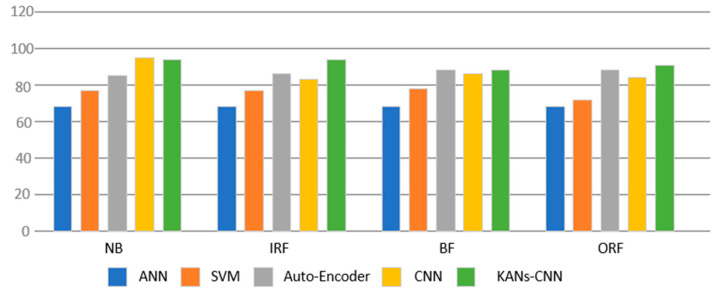
In comparative experiments with other methods, KANs-CNN achieved the highest performance in terms of average accuracy.

To further validate the effectiveness and feasibility of the diffusion network compared to traditional data generation networks, comparative experiments were conducted. The experiments were divided into two groups. The first group involved using CNN, SVM, Auto-Encoder, and KANs-CNN for fault diagnosis on a small sample-bearing dataset, with each method evaluated separately. The performance of different classifiers included: NB (Naive Bayes), a simple probabilistic classification method; IRF (Improved Random Forest), an ensemble learning method based on decision trees; BF (Bayesian Filtering), used for probabilistic sequence estimation and filtering; and ORF (Optimized Random Forest), which enhances the random forest algorithm for improved performance. The specific experimental results are illustrated in Figure 11, demonstrating that KANs-CNN, by employing learnable activation kernels to replace traditional linear activation functions, exhibits strong performance across various models.

**Figure 12:**
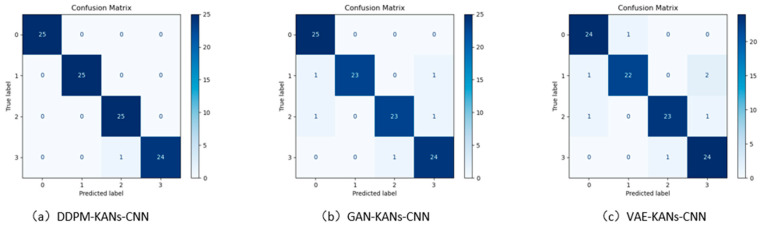
Comparison of confusion matrices for fault diagnosis accuracy among the DDPM, GAN, and VAE generative models.

The second group of comparative experiments utilized the small sample augmentation model DDPM alongside KANs-CNN, comparing it to other generative models such as GAN and VAE. All models employed the same original dataset for data generation, maintaining consistent sample sizes and integrating them into the original dataset. To ensure optimal performance for each model, the ratio of real to generated samples was adjusted to 1:1.5, with 40 original samples and 60 generated samples, to achieve the best fault diagnosis results. While the sample generation methods effectively alleviated the issue of sample scarcity, there may still be performance gaps between the generated and real data in fault diagnosis. Finally, KANs-CNN was used to classify the augmented datasets, with the confusion matrix displayed in Figure 12. The diagonal elements represent the number of correctly classified samples within each class, and the diagnostic accuracies for each operational state are detailed in Table 4.

### 3.2. Case 2

#### 3.2.1. Experiment Data of Case 2

To further assess the network’s effectiveness and generalization, the tool dataset from the PHM 2010 Tool Challenge was used. The spindle speed was set to 10,400 RPM, with a feed rate of 155 mm/min, radial cutting depth of 0.125 mm, and axial cutting depth of 0.2 mm. Data were collected at 20 kHz per channel, including cutting force, vibration, and AE signals. The dataset consists of 7 columns and 218,892 rows, with columns 4, 5, and 6 representing the vibration signals.

The dataset preprocessing included outlier handling, noise removal, and data normalization. The K Means clustering algorithm [39] was applied to categorize the fault types into four classes. The data was then transformed into three-channel RGB time-frequency maps using the discrete wavelet transform (DWT) and further processed with mean filtering. The resulting images were split into training, testing, and validation sets in a 3:1:1 ratio.

**Figure 13:**
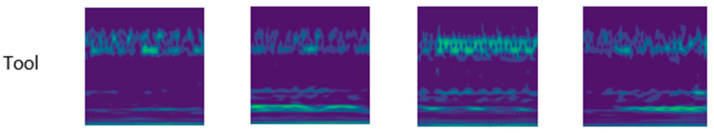
Time-frequency maps of the tool after wavelet transformation.

**Figure 14:**
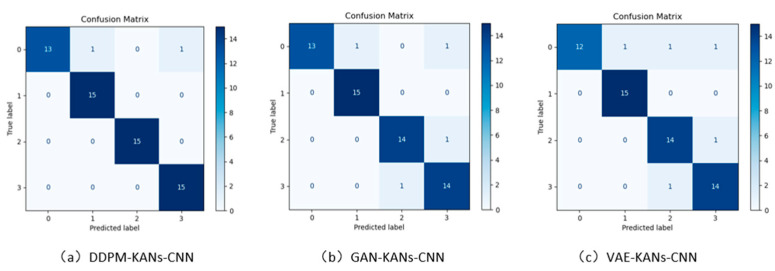
Comparison of confusion matrices for fault diagnosis accuracy among the DDPM, GAN, and VAE generative models.

To verify the effectiveness of the KANs-CNN fault diagnosis model in handling small sample data, the training set for one fault type comprised six samples, while the testing set included two samples. All data were processed into time-frequency maps using the wavelet transform, with the images for different fault types displayed in Figure 13. The experimental results are summarized in Table 5, and the confusion matrix is shown in Figure 14.

#### 3.2.2. Experiment Results of Case 2

Five representative existing methods were selected for comparison, including traditional CNN, MLP, SVM, GAN, and VAE. All methods utilized the small sample tool dataset, with data augmentation applied to the generative models, resulting in a total of 1.5 times the original data quantity, which was then mixed for the final fault diagnosis.

Through comparative experiments, the performance of each model was assessed based on key metrics, such as accuracy, precision, recall, and F1 score, to validate the advantages of KANs-CNN in handling small sample data. The experimental results indicated that KANs-CNN performed exceptionally well in fault diagnosis, particularly in scenarios with scarce samples, demonstrating its robust feature extraction and generalization capabilities. Detailed experimental results are presented in Table 6.

The KANs-CNN network demonstrated stronger fault diagnosis performance than traditional MLP models, even with small sample datasets, as illustrated in Figure 13. Particularly on the augmented dataset, KANs-CNN achieved an accuracy of 92.1% and an F1 score of 92.4%, significantly outperforming traditional methods. This indicates that KANs-CNN possesses a powerful capability for feature learning, enabling accurate identification of fault types in complex environments.

By integrating the denoising diffusion probabilistic model (DDPM) with KANs-CNN, the model reached top-tier performance in the diagnosis. This showcases the data generation capability of DDPM and its ability to extract rich mid-level information, allowing the model to perform well, even with larger datasets.

It is evident that the KANs-CNN fault diagnosis network outperforms traditional MLP models in diagnosing tool faults with smaller sample sizes. Moreover, DDPM consistently delivered better fault diagnosis results compared to VAE and GAN under the same sample augmentation conditions, highlighting the stability of data generation and the richness of feature information in the generated data.

In fault diagnosis, KAN-CNN combines the nonlinear activation of KAN and the feature extraction ability of CNN, effectively extracting deep and complex features from vibration signals, thereby improving diagnostic accuracy. FAN fuses features from different layers, ensuring effective aggregation of information and enhancing the model’s robustness. DDPM demonstrated strong data augmentation capabilities.

Currently, small sample fault diagnosis for rolling bearings faces challenges, such as high-dimensional feature extraction, nonlinear modeling, and a lack of labeled data. Future work could focus on optimizing data generation strategies, improving feature fusion accuracy, and exploring new deep learning models. Additionally, data augmentation is an effective solution to the small sample problem. In addition to conventional geometric transformations, methods such as noise injection, multi-class Generative Adversarial Networks (MC-GANs) for generating virtual samples, and time-frequency domain hybrid augmentation can be employed to further expand the dataset and improve the model’s robustness and accuracy.

## 4. Conclusions

The proposed KANs-CNN-based small sample fault diagnosis method for bearings combines the learnable activation functions of Kolmogorov–Arnold Networks (KANs) with CNN for feature extraction and classification. Experimental results demonstrate that this method performs exceptionally well in small sample scenarios, accurately capturing complex signal features while effectively avoiding overfitting. Compared to traditional methods, it achieves higher diagnostic accuracy and shows significant advantages under small sample conditions. As the number of newly generated samples increases within a certain range, the fault diagnosis accuracy also improves, reaching its peak at a ratio of 1:1.5. This indicates that the generated samples, when enhanced through the diffusion network, contain useful fault information that aids the fault diagnosis model in extracting critical fault details, thereby improving diagnostic accuracy. Training experiments show satisfactory accuracy in both bearing fault diagnosis and in diagnosing tool faults with fewer samples. This method provides valuable support for addressing the small sample issues in industrial equipment fault diagnosis.

## Figures and Tables

**Table 1:** Description of the CWRU Dataset.

Fault Type	Fault Size	Fault Position	Training Dataset for KANs-CNN	Label
Inner Race Fault	21 miles	-	10	0
Ball Fault	21 miles	-	10	1
Outer Race Fault	21 miles	centered@12:00	10	2
Normal	-	-	10	3

**Table 2:** Comparison of Accuracy Before and After Expansion.

Dataset	Bearing (%)				Expended Bearing (%)			
	Accuracy	Precision	Recall	F1 Score	Accuracy	Precision	Recall	F1 Score
Simple MLP	87.5	87.0	87.8	87.4	92.4	92.0	92.5	92.2
Conv KAN (2 Layer)	95.2	95.2	94.9	95.0	98.1	98.3	97.8	98.0
Conv KAN (4 Layer)	95.4	95.7	95.5	95.6	98.5	98.5	98.4	97.9
Conv KAN (6 Layer)	96.3	96.4	96.2	96.3	99.0	99.1	98.9	99.0

**Table 3:** Diagnostic results of different experiments with and without generated samples.

Experiment Number	Training Sample (Fault Types)		Training Sample (Normal Types)	Testing Samples	Average Accuracy (%)
	Real Sample	Generate Sample	Real Sample		
1	90	0	30	30	99.59
2	60	0	20	30	97.78
3	45	0	15	30	95.45
4	30	0	10	30	92.33
5	15	0	5	30	88.26
6	30	60	30	30	97.55
7	30	45	25	30	99.41
8	30	30	20	30	97.23
9	30	15	15	30	95.44
10	30	10	20	30	94.26
11	0	90	30	30	92.35
12	0	60	20	30	95.44
13	0	45	15	30	87.56
14	0	30	10	30	82.78
15	0	15	5	30	74.26

**Table 4:** Comparison of fault diagnosis data between different generative networks and DDPM.

Fault Diagnosis Model	DDPM-KANs-CNN	GAN-KANs-CNN	VAE-KANs-CNN
Accuracy	99.56%	97.12%	96.10%
Precision	99.15%	97.12%	96.05%
Recall	99.14%	97.03%	96.09%
F1 Score	99.45%	97.10%	96.01%

**Table 5:** Description of the classified PHM 2010 tool dataset.

Fault Type	Training Set	Test Set	Validation Set	Label
Early wear	24	8	8	0
Normal wear	24	8	8	1
Severe wear	24	8	8	2
Rapid wear	24	8	8	3

**Table 6:** Tool experiment comparison.

Dataset	Tool (%)				Expended Tool (%)			
	Accuracy	Precision	Recall	F1 Score	Accuracy	Precision	Recall	F1 Score
Simple MLP	78.7	78.1	78.4	72.3	84.5	82.0	82.4	82.2
KANs-CNN	87.2	87.2	87.9	87.0	93.1	93.3	92.5	92.9
GAN-KANs-CNN	-	-	-	-	93.5	93.3	93.9	94.3
VAE-KANs-CNN	-	-	-	-	91.1	91.9	91.0	91.9
DDPM-KANs-CNN	-	-	-	-	97.4	97.2	97.4	97.0

## Data Availability

The data presented in this study are available on request from the corresponding author. The raw/processed data needed to reproduce these findings cannot be shared publicly at this time, as they are also part of an ongoing study.
